# The Impacts of Slc19a3 Deletion and Intestinal SLC19A3 Insertion on Thiamine Distribution and Brain Metabolism in the Mouse

**DOI:** 10.3390/metabo13080885

**Published:** 2023-07-26

**Authors:** Anita Wen, Ying Zhu, Sook Wah Yee, Brian I. Park, Kathleen M. Giacomini, Andrew S. Greenberg, John W. Newman

**Affiliations:** 1 Department of Nutrition, University of California, Davis, CA 95616, USA; 2 West Coast Metabolomics Center, Genome Center, University of California, Davis, CA 95616, USA; 3 Gerald J. and Dorothy R. Friedman School of Nutrition Science & Policy, Tufts University, Boston, MA 02111, USA; 4 Jean Mayer USDA Human Nutrition Research Center on Aging, Tufts University, Boston, MA 02111, USA; 5 Department of Bioengineering and Therapeutic Sciences, University of California, San Francisco, CA 92521, USA; 6 USDA Western Human Nutrition Research Center, Davis, CA 95616, USA

**Keywords:** thiamine, thiamine transporter 2, THTR2, brain metabolism, metabolomics

## Abstract

The Thiamine Transporter 2 (THTR2) encoded by *SLC19A3* plays an ill-defined role in the maintenance of tissue thiamine, thiamine monophosphate, and thiamine diphosphate (TDP) levels. To evaluate the impact of THTR2 on tissue thiamine status and metabolism, we expressed the human *SLC19A3* transgene in the intestine of total body *Slc19a3* knockout (KO) mice. Male and female wildtype (WT) and transgenic (TG) mice were fed either 17 mg/kg (1×) or 85 mg/kg (5×) thiamine hydrochloride diet, while KOs were only fed the 5× diet. Thiamine vitamers in plasma, red blood cells, duodenum, brain, liver, kidney, heart, and adipose tissue were measured. Untargeted metabolomics were performed on the brain tissues of groups with equivalent plasma thiamine. KO mice had ~two- and ~three-fold lower plasma and brain thiamine levels than WT on the 5× diet. Circulating vitamers were sensitive to diet and equivalent in TG and WT mice. However, TG had 60% lower thiamine but normal brain TDP levels regardless of diet, with subtle differences in the heart and liver. The loss of THTR2 reduced levels of nucleic acid and amino acid derivatives in the brain. Therefore, mutation or inhibition of THTR2 may alter the brain metabolome and reduce the thiamine reservoir for TDP biosynthesis.

## 1. Introduction

Thiamine, which is found in meats, grains, beans, and fortified products, is an essential nutrient for cellular functions. In the cell, this vitamin is converted to various derivatives including thiamine monophosphate (TMP) and thiamine diphosphate (TDP). Thiamine and TMP are influenced by recent exposure and are the main forms in plasma available for tissue uptake [1,2], whereas intracellular TDP is a cofactor for several metabolic enzymes involved in sugar, amino acid, and fat metabolism [3]. TDP levels are regulated [4] and often used as a clinical status biomarker for deficiency either directly or via functional assays in red blood cells (RBC) [1]. In addition to energy metabolism, this vitamin has downstream effects in cellular oxidation, nucleotide production, and neurotransmitter functions [5,6]. Insufficient thiamine is associated with reduced energy and greater oxidative stress and inflammation. As charged compounds, thiamine and its derivatives cross membranes through the actions of multiple active transporters, several of which are co-localized, underscoring thiamine’s importance for cellular functions [7,8,9]. Various low-affinity high-capacity nonspecific transporters, including the organic cation transporters (OCTs) [10,11,12] and multidrug and toxic compound exclusion efflux transporters (MATEs) [13], can transport thiamine. Specific thiamine vitamer transporters include thiamine transporters (THTR1 and 2) [14,15,16,17,18], the thiamine transporter *SLC35F3* [19], the reduced folate carrier 1 (RFC1) *SLC19A1* reported to transport TMP and TDP [20,21], the mitochondrial TDP carrier *SLC25A19* [22,23], and the intestinal TDP transporter *SLC44A4* [24,25]. While genetic ablation or dysfunction of these transporters lead to differentiating phenotypes [17,23,26,27], the relative contribution of the various transporters on thiamine uptake into different tissues is currently unclear. Both THTRs are well known for thiamine absorption in the gut. THTR1 (Km = 2.5 µM) is localized on both the luminal and basement membranes participating in absorption and transfer to the blood. The mRNA expression of THTR2 is ~2.5 higher than THTR1 in the duodenum [28]. THTR2 (Km = 27.1 nM) is expressed on the luminal brush border [18]. Previous studies investigating mice with whole body deficiency of THTR2 and in vitro knockdown of THTR2 in Caco-2 cells indicated that this transporter was important for thiamine intestinal absorption [15,18]. This study focuses on the effect of the *SLC19A3* gene product THTR2 on thiamine status in multiple tissues.

Over the past decade, marketed drugs have been identified to inhibit THTR2 including metformin, trimethoprim, and fedratinib [28,29,30,31]. During the 2011–2012 fedratinib phase II and phase III clinical trials, eight subjects experienced encephalopathy, a potential symptom of thiamine deficiency. Although later in vitro studies showed that fedratinib inhibited THTR1 and THTR2 [28,29,32] and that a 500 mg dose was predicted to inhibit intestinal absorption [30], the cause of encephalopathy in the trials was inconclusive or dismissed as unrelated to fedratinib intake [31]. As of today, fedratinib has reached the market with a black box label informing that the drug can potentially cause fatal encephalopathy and recommends thiamine monitoring [33]. Although it is uncertain if fedratinib causes Wernicke’s encephalopathy, these trials highlight the potential for drug–nutrient interaction and questions if potent THTR2 inhibitors are a safety concern for drug-induced localized deficiency especially for populations at risk for thiamine deficiency. Thus, understanding of THTR2 contribution to thiamine uptake in various tissues would gauge the possible impact of these inhibitors.

To probe the contribution of THTR2 for thiamine status in various organs, we took advantage of a transgenic mouse model created to explore drug–THTR2 interactions in the gut. For the generation of the transgenic mice (*Slc19a3*^−/−; *SLC19A3*int^; TG), we inserted the human THTR2 gene *SLC19A3* driven by the villin promoter into the THTR2 knockout (*Slc19a3*^−/−^; KO) for intestine-specific expression. Murine and human THTR2 have differential affinity for inhibitors. For example, metformin and fedratinib are at least nine times more potent for the human THTR2 than mouse THTR2 [28]. In this study, we show that the transgenic line expressing the human THTR2 has functional thiamine gut absorption and similar plasma concentrations as wildtype mice (WT). This observation allowed us to explore the impact of deficient THTR2 expression on thiamine status in various tissues including the brain, heart, adipose, liver, and kidneys under two diets with a five-fold difference in thiamine hydrochloride (HCl) levels. Here, we show that THTR2 helps maintain thiamine status in the brain and that ablation reduces the concentration of a variety of small cations including derivatives of nucleic acids and amino acids in this tissue. 

## 2. Materials and Methods

### 2.1. Generation of Human THTR2 Expressing TG Mice

The human THTR2 in the TG mice was inserted under control of the villin promoter/enhancer. The purified human THTR2 transgene was constructed in the pBS KS Promoteur Villin MES SV40 polyA plasmid. After digestion with Sal I, the 10-kb transgene was isolated. Purchased from Jackson lab as embryos [Strain #: 017343] [15], the THTR2 KO male mice were crossed with B6D2F1 females to produce zygotes used for pronuclear injection of the transgene. At the Beth Israel Deaconess Transgenic Core Facility, standard pronuclear injection methods [34] were used to generate the transgenic mice. Littermates were crossed after the F0 generation and were screened for the presence of the transgene in the tail genomic DNA via conventional PCR. Littermates consisted of WT, KO, and TG mice.

### 2.2. Quantitative Polymerase Chain Reactions

RNA expression of THTR2 and THTR1 were analyzed in the jejunum and brain. Tissue was mechanically homogenized in TRIzol (Invitrogen, Waltham, MA, USA) by Tissue lyserII (Qiagen, Germantown, MD, USA), and total RNA was extracted with RNeasy Mini columns (Qiagen) according to the manufacturer’s instructions. Reverse transcription was performed using a High-Capacity cDNA Reverse Transcription Kit (Applied Biosystems, Waltham, MA, USA). Quantitative real-time PCR (qPCR) was performed using SYBR Green (Applied Biosystems) on an Applied Biosystems 7300 Real-Time PCR System. 

### 2.3. Western Blotting

Due to variability of the villin promoter on expression [35], hTHTR2 protein expression was evaluated in various tissues. Tissue extracts were prepared in RIPA lysis buffer (Thermo Fisher, Waltham, MA, USA) with dissolved Pierce protease inhibitor tablets (Thermo Fisher) followed by homogenization. After centrifuging the homogenates at 15,000× *g* and at 4 °C for 15 min, supernatants were collected, and the protein content was determined by Pierce BCA Protein Assay Kit (Thermo Fisher). Samples of 20 μg of protein were electrophoresed on precast Mini-protean TGX gels with 4–20% polyacrylamide (Bio-Rad, Hercules, CA, USA), transferred to a low-fluorescence PVDF membranes (Thermo Scientific), and then blocked in Tris-buffered saline with 0.1% Tween 20 (TBS-T) supplemented with 5% non-fat dry milk at room temperature for 1 h. Membranes were incubated overnight at 4 °C with the 1:1000 dilutions of rabbit anti-*SLC19A3* primary antibodies (1:1000, Sigma-Aldrich, St. Louis, MO, USA) in TBS-T supplemented with 5% non-fat dry milk, then washed with TBS-T and incubated with 1:2000 dilutions of secondary horse radish peroxidase conjugated goat anti-rabbit IgG (Cell Signaling Technologies, Danvers, MA, USA). Signals were detected using the enhanced chemiluminescence SuperSignal West Femto Maximum Sensitivity Substrate detection kit (Thermo Fisher).

### 2.4. Thiamine Feeding Study

The feeding study was conducted at the Jean Mayer U.S. Department of Agriculture Human Nutrition Research Center on Aging in accordance with guidelines and regulations approved by the Institutional Animal Care and Use Committee of Tufts University. WT, TG, and KO littermates were cohoused and fed on an AIN93G-based high thiamine diet containing 85 mg/kg thiamine HCl (Research Diets, New Brunswick, NJ, USA) until 15–16 weeks of age. After a baseline body composition measurement via magnetic resonance imaging (EchoMRI, Houston, TX, USA), animals were individually housed and randomized into two diet interventions: a standard 17 mg/kg thiamine HCl (1×) or 85 mg/kg thiamine HCl (5×) diets for 5 weeks (*n* = 12/group; 6 female, 6 male). KO were not viable on the 1× diet and thus the KO mice on that diet were omitted from the study. For 4 weeks, body weight and food intake were monitored weekly and body composition was determined every two weeks. After 2 h of fasting on the last day, mice were anesthetized by isoflurane and euthanized by exsanguination and cervical dislocation. Blood was collected in EDTA-coated tubes and centrifuged at 2000× *g* and 4 °C for 15 min for RBC and plasma separation. The two fractions were stored at −70 °C until analysis. Duodenum, brain, kidney, liver, heart, and retroperitoneal fat were snap-frozen and kept at −70 °C until analysis. Six animals from each of the five groups were analyzed for thiamine vitamers.

### 2.5. In Vivo THTR2 Inhibition Study

MuriGenics, Inc. (Vallejo, CA, USA) performed a thiamine–trimethoprim interaction study using 9- to 11-week-old (~20–25 g) FVB mice (*n* = 11) from Charles River Laboratories (Wilmington, MA, USA). Prior to the intervention, mice were fed a low thiamine diet (5 mg/kg) for one week followed by a thiamine-deficient diet (0 mg/kg) for the second week. The mice were then fasted for 16 h before treatment. On the day of the study, the mice were orally gavaged with either 2 mg/kg thiamine (*n* = 6; 3 female, 3 male) or a combination of 2 mg/kg thiamine and 61.5 mg/kg trimethoprim (*n* = 5; 3 female, 2 male). The brains were collected 3 h post intervention for thiamine vitamer analysis.

### 2.6. Plasma Thiamine Analysis

Plasma thiamine was quantified using ultra-performance liquid chromatography tandem mass spectrometry (UPLC-MS/MS). Briefly, in 1 mL polypropylene 96-well plates (Nunc, Waltham, MA, USA), plasma (10 µL) was spiked with 10 µL of 1 µM thiamine-d3 (Medical Isotopes, Pelham, NH, USA) as an internal standard (ISTD) and diluted with 80 µL of chilled acetonitrile containing 0.1% acetic acid and 0.55 mM ammonium acetate. The plates were shaken for 1 min and centrifuged at 2247× *g* and 4 °C for 15 min. Using 0.2 µm PVDF filters plates (Agilent Technologies, Santa Clara, CA, USA), supernatants were filtered under the same centrifugal force and temperature for 2 min. Filtered samples (10 µL) were then injected on a H-class Acquity UPLC (Waters Corp, Milford, MA, USA) and separated with a 2 × 150 mm, 3 µm Luna Silica column attached to a 0.5 µm depth × 0.004 in internal diameter in-line KrudKatcher filter (Phenomenex, Torrance, CA, USA) at 35 °C. Mobile phases consisting of 0.1% acetic acid in water (A) and 0.1% acetic acid in acetonitrile (B) were flowed at 0.4 mL/min using the following gradient. The initial conditions of 2%B held for 0.5 min were increased to 13%B at 2.5 min and 100%B at 3.0 min. After a 1.5 min hold, the column was re-equilibrated for 2 min at 2%B. All the solvents and water were LC/MS-grade from Fisher Scientific (Pittsburgh, PA, USA), while solvent additives were from Sigma-Aldrich. On an API 4000 QTRAP (Sciex, Framingham, MA, USA), precursor-product mass transitions for thiamine (265.0 > 122.1 *m*/*z*) and thiamine-d3 (268.0 > 125.0 *m*/*z*) were monitored in positive mode. The temperature, ion spray voltage, gas1, and gas2 were set at 550 °C, 4500 V, 40 psi, and 40 psi, respectively. Thiamine was quantified in MultiQuant 3.02 (Sciex) using peak area ratios against a minimum 9-point calibration curve. The lowest calibration point contained thiamine-d3 to correct for background impurities. Two method blanks, three pooled mouse plasma, and three pooled human plasma were processed with experimental samples. 

### 2.7. RBC and Tissue Thiamine Vitamer Analysis

For the feeding study, thiamine, TMP, and TDP in RBCs, brain, liver, heart, retroperitoneal fat, duodenum, and kidney were measured by UPLC-MS/MS. Briefly, ~15 mg RBCs, ~75 mg brain, ~35 mg fat, ~40 mg liver, ~25 mg heart, ~25 mg duodenum, or ~25 mg kidney, were enriched with 10 µL of a water containing ^13^C3 thiamine (Cerilliant Corp, Round Rock, TX, USA), and TDP-d3 (Toronto Research Chemicals, Toronto, ON, Canada). For the THTR2 inhibition study, only 30 mg of brain was used. Methanol (200 µL) was added for protein precipitation before homogenization on a 2010 Geno/Grinder vertical ball mill (SPEX SamplePrep, Metuchen, NJ, USA) for 1 min at 1300 rpm using a stainless-steel bead. The samples were centrifuged at 20,000× *g* and 4 °C for 15 min, and the resulting supernatants were dried under vacuum. After reconstitution in 0.5% aqueous formic acid, the extracts were filtered through 0.2 µm PVDF filter plates (Agilent Technologies) by centrifugation at 2247× *g* for 2 min. Brain, liver, heart, duodenum, and kidney samples were diluted 5-fold with 0.5% aqueous formic acid to capture analytes within their analytical curves. Ten microliters of extracts were injected on a Waters H-class Acquity UPLC connected to an API 4000 QTRAP (Sciex) mass spectrometer. Using a modified published method [36], the analytes were separated on a 4.6 × 100 mm, 3 µm Gemini NX-C18 column attached to a SecurityGuard with a 3 × 4 mm Gemini NX-C18 cartridge (Phenomenex, Torrance, CA, USA) at 15 °C. Mobile phases consisting of 10 mM ammonium bicarbonate in water (A) and methanol (B) flowed at 0.6 mL/min for the following gradient. Initial conditions of 0%B held for 0.5 min were increased to 50%B at 3.5 min and 95%B at 4.0 min. After a 2 min hold, the column was re-equilibrated for 3 min at 0%MPB. Analytes were detected by positive mode electrospray ionization using multi-reaction monitoring of precursor–product mass transitions for thiamine (265.0 > 122.1 *m*/*z*), TMP (345.0 > 122.1), and TDP (425.1 > 122.2), and their internal standards 13C3-thiamine (268.0 > 122.0 *m*/*z*) and TDP-d3 (428.1 > 125.2 *m*/*z*). For source parameters, temperature, ion spray voltage, gas1, and gas2 were set at 650 °C, 2000 V, 40 psi, and 40 psi, respectively. Quantification was performed using internal standard methodology and 9-pt calibration curves prepared daily with samples by serial dilution of primary stocks stored at −80 °C. A master mix containing 100 µM thiamine (Cerilliant, Round Rock, TX, USA), TMP chloride dihydrate (Sigma-Aldrich, St Louis, MO, USA), and TDP (Sigma-Aldrich) in methanol and a ISTD mix containing ^13^C3-thiamine and TDP-d3 in water was diluted with 0.5% formic acid to result 2500 nM analytes, 30 nM ^13^C3-thiamine, and 400 nM TDP-d3 for diluted samples or 2500 nM analytes and 150 nM ^13^C3-thiamine and 2000 nM TDP-d3 for undiluted samples. The mix was then serially diluted 2–5x fold per step with ISTD in 0.5% formic acid to result the linear range of 0.5–2500 nM. A calibration solution containing only ISTDs was used as a zero point to correct for any native background in isotopically labeled compounds. Since isotopically labeled TMP was not commercially available, we evaluated TMP correction using either ^13^C-thiamine or TDP-d3. Using ^13^C-thiamine-based corrections yielded vitamer ratios more similar to reported fluorometric-based methods and were selected for reporting. Our vitamer values for plasma and tissue levels in WT mice were comparable to a prior study on total vitamers [12]. Samples were analyzed using MultiQuant 3.02 (Sciex) for quantification. Each analytical batch contained 2–3 method blanks and one sample in triplicate or duplicate per matrix processed in the batch. See Appendix A and Supplementary Table S1 for assay performance on blanks and replicates.

### 2.8. Brain Metabolomics

To further investigate the effects of genotype on brain thiamine metabolism, we performed untargeted metabolomic analyses on brain samples from mice with similar circulating thiamine vitamer levels, namely WT and TG fed the 1× diet and KO mice fed the 5× diet. Brain cortices of 12 mice/group (6 male, 6 female) were analyzed at the UC Davis West Coast Metabolomics Center (University of California, Davis, CA, USA). The samples were processed via established methods [37] using gas chromatography tandem time of flight (GC-TOF) and liquid chromatography quadrupole time of flight (LC-QTOF) mass spectrometry. 

Briefly, 4 mg brain cortex was ground, and proteins were removed via protein precipitation. Supernatants were split into aliquots for GCMS and LCMS analyses and dried under vacuum. Residues were reconstituted in 50:50 (*v*/*v*) acetonitrile: water. After centrifugation, the supernatant was dried under vacuum and stored at 20 °C until further processing. For GCMS on the day of analysis, residues were mixed with methoxyamine HCl in pyridine followed by treatment with N-methyl-N-trimethylsilyl-trifluoroacetamide and spiked with fatty acid methyl esters as a retention time reference. Metabolites were separated on a Restek Rtx-5Sil MS w/Integra-Guard column (0.25 mm, 30 m, Restek Corp, Bellefonte, PA, USA) in an Agilent Technologies 7890A GC, interfaced with a Leco Pegasus IV time of flight (Leco Corportaion, St. Joseph, MI, USA). For LCMS on the day of analysis, residues were reconstituted in 20% acetonitrile (100 µL) containing 35 deuterated ISTDs and 1-cyclohexyl-ureido, 3-dodecanoic acid (Sigma-Aldrich). Metabolites were separated on 1260 Infinity HPLC (Agilent Technologies) using a 2.1 × 150 mm, 1.7 µm Acquity UPLC BEH amide column (Waters Corp) attached to a 2.1 × 5 mm, 1.7 µm Acquity BEH amide guard column, interfaced with a QTOF 6600 (Sciex) mass spectrometer. 

The GC-MS/MS peak height outputs were processed using BinBase [38], while LC-MS/MS peak height outputs were processed by MS-DIAL [39]. MassBank of North America and NIST17 libraries were used to annotate features based on retention times, parent masses, and MS/MS spectra. Duplicate peaks, ion adducts, and isotopes were detected by the MS feature list optimizer, MS-FLO [40]. To compare samples, data were normalized by peak height/sum of ISTD × average sum of deuterated ISTD in all samples; this normalized data will be herein referred to as ‘raw’ data. PubChem Identifier Exchange Service was used to obtain SMILES and Pubchem chemical IDs from the InChIKeys. 

### 2.9. Statistics 

Prior to analysis, datasets were evaluated for outliers and normality and appropriate steps were taken to clean and analyze the dataset as described below. Parametric and nonparametric approaches were used as appropriate. Mean testing, mixed effects modeling, and partial least squares discriminant analyses (PLSDA) were performed in JMP Pro 16 (SAS Institute, Carry, NC, USA). Random Forest (RF) analyses were performed in R version 4.2.2.

#### 2.9.1. Group Characterizations

Body weight, food intake, fat mass, and fat mass percentage were evaluated by sex using mixed model regressions with group and week by sex. Tissue weights of the liver, heart, kidneys, fat, and brain were compared separately by sex using ANOVA and Tukey’s Honest Significant Difference post hoc tests.

#### 2.9.2. Feeding Study Thiamine Vitamer Analysis

Of the samples analyzed, one plasma sample from a 5× WT male mouse diet failed during extraction and was excluded from the group comparisons. Outliers identified in untransformed data by a robust fit outliers quartile test were treated as missing. Data normality was evaluated by Shapiro–Wilk tests and Log or Johnson transformed if required. Two data points, the above-mentioned plasma thiamine, and a single kidney TMP outlier from a 1× TG female mouse, were imputed using multivariate imputations. To compare the vitamers among the five groups, ANOVA and post hoc Tukey’s tests were performed. To evaluate variables affecting thiamine status between WT and TG mice, linear regressions were performed with genotype, sex, and plasma thiamine concentration and their interactions as fixed effects. Models were simplified in a stepwise fashion removing variables if the coefficients were not significant (*p* > 0.05). To test the impact of diet on tissue vitamer status, least square regressions were repeated, replacing plasma thiamine with diet as a categorical variable.

#### 2.9.3. THTR2 Inhibition Study Thiamine Vitamer Analysis 

One mouse from the trimethoprim group died post-treatment and was excluded from analysis. Mann–Whitney U-tests were used to identify treatment effects on brain vitamers.

#### 2.9.4. Metabolomics Data Pre-processing 

LC and GC metabolomic data from animals with equivalent plasma thiamine concentrations (i.e., 1× WT, 1× TG and 5× KO) were combined and analyzed in aggregate. Of the 1430 features found, 406 were annotated by library matches and separated from unknowns. Of 14,616 data points, 172 outliers (0.012%) were detected by robust fit Huber’s test and treated as missing in the annotated dataset. For each metabolite, data normality was assessed using Shapiro’s test for untransformed, log-, square root-, and Johnson- transformed data. If normality was not achieved, Box–Cox transformation was also attempted. Normality was achieved for 97% of the metabolites. Sixty-six duplicate metabolites with the higher %CV in pooled samples were removed resulting in a final dataset of 340 unique known metabolites. Data were then mean centered and scaled to achieve normal distribution with a mean of 0 and variance of 1. Prior to modeling, missing values were imputed via the singular variable decomposition method in JMP.

#### 2.9.5. Chemical Enrichment Analysis 

To evaluate enrichment of chemical classes, ChemRICH was used to classify metabolites and to cluster them by chemical class using *p*-values from the beta coefficients for genotype effect from regression models as well as fold changes of raw peak heights as input variables [41].

#### 2.9.6. Multivariate Analyses

Metabolites differing between experimental groups were identified, and discriminating variables were scored using PLSDA and RF. To find differentiating metabolites between the three comparisons (i.e., WT vs. TG, WT vs. KO, and KO vs. TG), PLSDA was performed using leave-one-out cross validation. A cumulative Q^2^ > 0.4 was considered to indicate full group discrimination, and variable importance in projection (VIP) scores >1.0 indicated discriminating variables. In addition, variable selection via random forest (RF) was performed using R package *ranger* to build 5000 trees without sample replacement [42,43]. Package *tuneRanger* was used to determine the hyperparameters including minimum node size, number of variables considered at splitting points (mtry), and the sample fraction used to build the trees [44]. Importance values and the *p*-values from 100 permutations were collected. A PLSDA-RF ensemble scoring system was created combining the two variable selection methods to identify top differentiating candidate metabolites as follows: (1) PLSDA VIP scores <1, 1–1.249, 1.25–1.49, 1.5–1.749, and >1.75 were assigned 0, 0.25, 0.5, 0.75, and 1, respectively; (2) permuted RF *p*-values <0.05, 0.051–0.1, 0.101–0.15, 0.151–0.2, and >0.2 were scored as 1, 0.75, 0.5, 0.25, and 0, respectively. If the importance value was 0 or negative, then the RF score was automatically made 0; (3) the PLSDA and RF scores were summed for a total score between 0 and 2.

## 3. Results

### 3.1. Expression of Human THTR2, Mouse THTR2, and Mouse THTR1

Evidence of THTR2 ablation, intestine-specific human THTR2 expression, and mouse THTR1 responses to genetic manipulation was obtained. qPCR showed that TG and KO had absent or lower expression of THTR2 in the brain and jejunum compared to WT (Supplementary Figure S1A,B). There were no differences in THTR1 expression among WT, TG, and KO in the brain and jejunum. Antibodies directed against the THTR2 protein produced significant staining of a slow running protein smear in intestinal samples, but not other tissues (Supplementary Figure S1C). While the staining protein appeared substantially larger than the theoretical ~70 kD transgenic protein, THTR2 has both serine/threonine phosphorylation and glycosylation sites and is known to undergo post-translational glycosylation [45], which commonly result in a smeared signal in SDS-PAGE [46]. 

### 3.2. Characterization of Groups of Mice

For males, there were no differences in body weight, food intake, body composition, and the tissue weights of the liver, heart, kidney, retroperitoneal fat, and brain among the five groups of mice (Supplementary Figure S2). In females, there were no differences in food intake or tissue weights. The regression model for body weight suggested a weak group effect (*p* = 0.051) and Tukey post hoc analysis indicated that the 5× WT were heavier than the 5× KO mice. However, this weight difference was not substantial as KO/WT ratios were 0.89 at week 0 and 0.89 prior to euthanasia. In addition, female WT in the 1× diet had greater %fat compared to the 1× TG and 5× KO mice. The (1× TG)/(1× WT) and (5× KO)/(1× WT) %fat ratios were ~0.67 and ~0.58. However, further analysis using 33 WT, 36 TG, and 18 KO mice under the 5× diet since birth showed no difference in body weight and body composition.

### 3.3. Impact of THTR2 on Thiamine Distribution 

The KO mice did not survive on the 1× diet. Plasma collected from a single deceased KO had 8.3 nM thiamine, ~25× lower than the WT on the same diet. Thiamine vitamers measured in plasma, RBCs, duodenum, brain, liver, kidney, heart, and retroperitoneal fat are reported in Supplementary Table S2. The vitamers were compared among the five groups of viable mice. Experimental variables influenced thiamine vitamer levels in the plasma, RBC, duodenum, kidney, liver, and brain, with the strongest genotype-specific effects on the brain thiamine status. 

**Figure 1:**
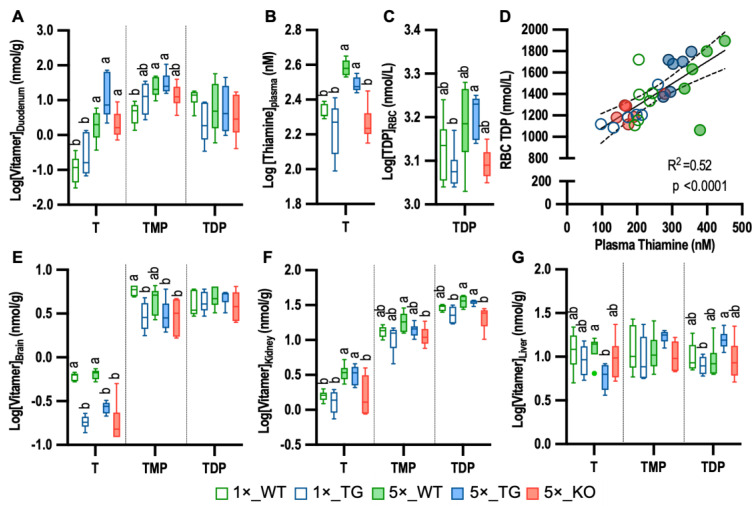
Impact of 17 mg/kg (1×) or 85 mg/kg (5×) thiamine HCl diets on thiamine vitamers in tissue and blood compartment of wildtype (WT), Slc19a3^−/−; SLC19A3int^ (TG), and Slc19a3^−/−^ (KO) mice (*n* = 6/grp). (**A**) Duodenum levels; (**B**) plasma levels; (**C**) RBCs levels; (**D**) plasma vs. RBC level correlation; (**E**) brain levels; (**F**) kidney levels; (**G**) liver levels. Diet x genotype group comparisons for each vitamer were performed by ANOVA with post hoc Tukey test. For each vitamer, means not sharing letter annotations differ between groups. Boxes without letters indicate equivalent levels among groups. One plasma sample in the 5×_WT group was excluded from plots. T = Thiamine; TMP = thiamine monophosphate; and TDP = thiamine diphosphate.

To determine the effect of the transgene expressed in the intestine, vitamers were compared in the duodenum and circulation. The TG mice were comparable to the WT mice for all three vitamers in the gut (Figure 1A), with the 5× diet increasing intestinal thiamine by 3.5-fold in WT and 5.5-fold in TG. While the KO mice had equivalent intestinal thiamine compared with the other two 5× diet groups, they had lower plasma thiamine level; their plasma thiamine and RBCs TDP were equivalent to the WT and TG mice on the 1× diet. Plasma thiamine and RBC TDP were similar between WT and TG groups on equivalent diets (Figure 1B,C). The 5× diet increased plasma levels ~1.7-fold over the 1× diet in both the WT and TG mice. While the TDP level of RBC in WT did not differ between the two diets and could not be distinguished from the TG on either diet, the 5× diet increased RBC TDP ~1.3-fold over the 1× diet in TG mice. Plasma and RBCs thiamine vitamers did not differ by sex in any group. Moreover, plasma thiamine was strongly correlated with RBC TDP levels (R2 = 0.52, *p* < 0.0001; Figure 1D), a common indicator of thiamine status.

Brain thiamine concentrations were ~3x lower in the TG (0.23 ± 0.06 nmol/g; *p* < 0.0001) and KO (0.21 ± 0.15 nmol/g; *p* < 0.0001) groups compared to the WT group (0.63 ± 0.07 nmol/g). Notably, the 5× diet did not rescue the brain thiamine levels in the absence of THTR2 expression. WT and TG brain thiamine vitamer concentrations were not influenced by dietary thiamine (Figure 1E). For TMP, diet did not influence brain levels in any genotype, but the KO group had 1.5x-fold lower levels than the WT mice in the 5× diet. The active cofactor TDP did not differ among the five groups. Similarly, acute THTR2 inhibition with trimethoprim reduced thiamine by 57% (*p* = 0.038) in the brain but had no effects on TMP and TDP (Supplementary Figure S3).

In contrast to the brain, renal thiamine was strongly influenced by diet (Figure 1F). Specifically, the WT and TG groups had comparable thiamine levels in both diets. The 5× diet increased thiamine 2.3-fold over the 1× diet (3.40 ± 1.3 vs. 1.49 ± 0.39 nmol/g, respectively, *p* < 0.0001) in WT and TG mice. While the diet did not change renal TDP in the WT groups, the TG group had 1.5× higher TDP in the 5× diet (34.88 ± 2.53 nmol/g) than the 1× diet (23.49 ± 6.23 nmol/g, *p* = 0.001). KO mice had lower renal thiamine, TMP, and TDP compared to the WT on the 5× diet and were comparable to the WT and TG on the 1× diet.

In the liver, the TG group on the 5× diet had 2x lower thiamine than the WT on this diet (*p* = 0.0058), while no differences were observed on the 1× diet (Figure 1G). The TG group showed 1.9-fold higher TDP in the 5× diet than in the 1× diet. No differences in heart or fat thiamine vitamers were detected among the five groups (Supplementary Figure S4).

### 3.4. Impact of Plasma Thiamine, Genotype, and Sex on Tissue Thiamine Levels

Dietary thiamine greatly influenced plasma thiamine concentrations (Supplementary Table S3). To control for dietary intake and absorption, linear regression models were built using plasma thiamine concentrations, genotype, and sex as fixed effects with all WT and TG animals (Table 1). The β coefficients in Table 1 show the relationships between the predictor variable (genotype, plasma thiamine, or sex) and the response variable (vitamer level) in the regression model. β coefficient magnitudes cannot be compared between vitamers due to vitamer concentration normality transformations. No significant fixed effect interactions were observed. After adjusting for genotype and sex, the plasma thiamine concentration was positively correlated with RBCs TDP, brain TDP, fat thiamine, heart TDP, and all three vitamers of kidney. Inspecting genotype impacts on tissue vitamer levels, after adjusting for plasma concentrations and sex, the TG genotype showed lower levels of brain thiamine and TMP, heart TDP, and liver thiamine. Moreover, controlling for plasma thiamine concentrations and genotype, male mice had greater brain thiamine and lower renal TDP than female mice.

### 3.5. Brain Metabolomic

**Figure 2:**
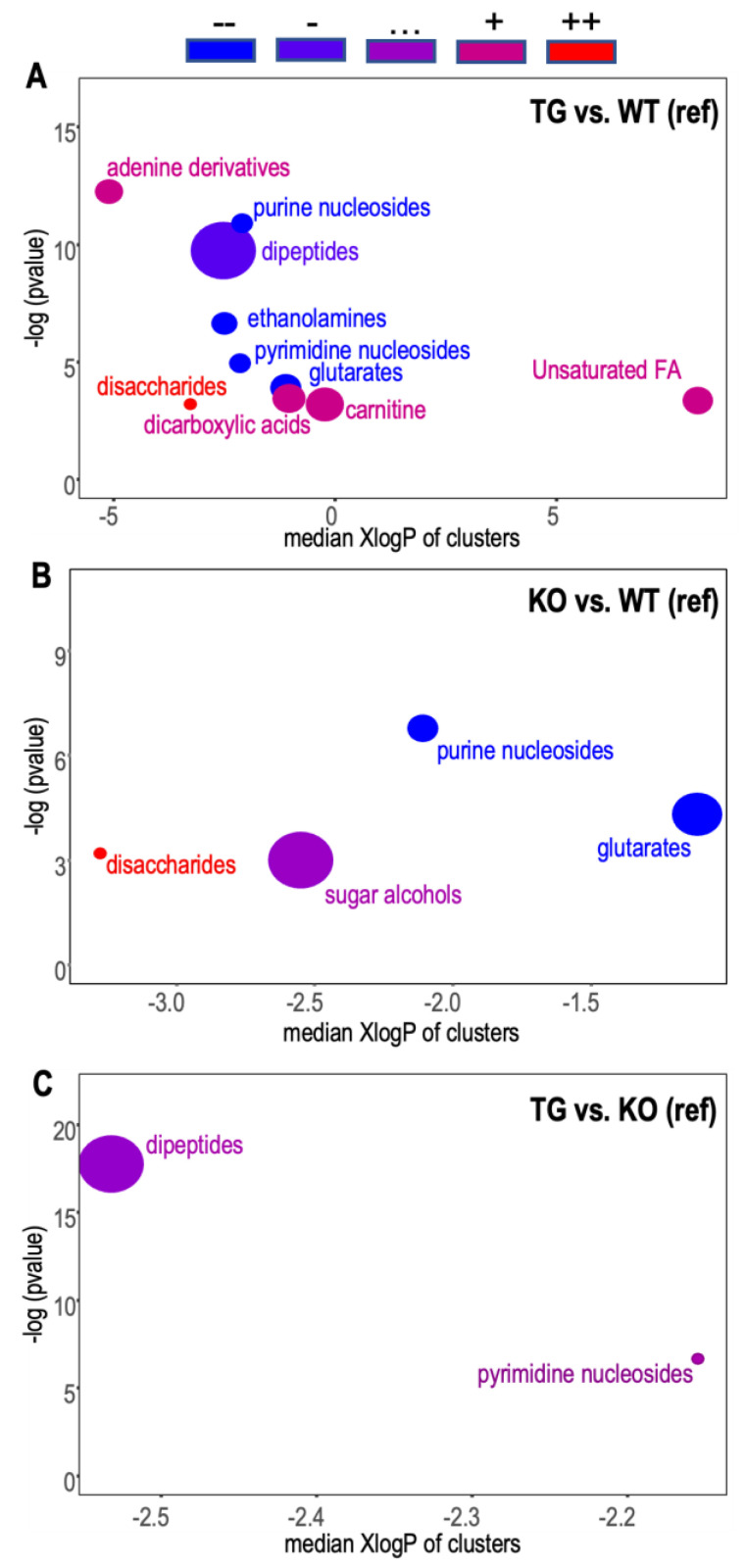
Comparisons of chemical class enrichment of brain tissues from wildtype (WT), Slc19a3^−/−; SLC19A3int^ (TG), and Slc19a3^−/−^ (KO) mice with equivalent plasma thiamine concentrations. (**A**) WT vs. TG; (**B**) WT vs. KO; (**C**) KO vs. TG. The chemical enrichment statistic was calculated by the Kolmogorov–Smirnov test in ChemRICH [41] using mean heights for directionality and *p*-value for genotype comparisons (*n* = 12/grp) obtained from mixed model regressions of metabolite peak heights adjusted for sex and genotype x sex interactions. Structural classes are shown if significantly enriched (*p* < 0.05). The chemical cluster node size is determined by the number of metabolites within the class. Node color depends on the ratio of significantly increased to decreased metabolites, indicating directionality of change from the reference group: blue = decreased; purple = bidirectional; red = increased.

From the ChemRICH analyses, both TG and KO showed lower levels of nucleosides and glutarates and an increase in disaccharides compared to WT (Figure 2A,B). The TG group showed additional depletion in ethanolamides and weak enrichment in carnitine, dicarboxylic acids, and unsaturated fatty acids. The KO and TG mice had very similar chemical class profiles, with identified significant differences showing no average directional changes (Figure 2C).

**Figure 3:**
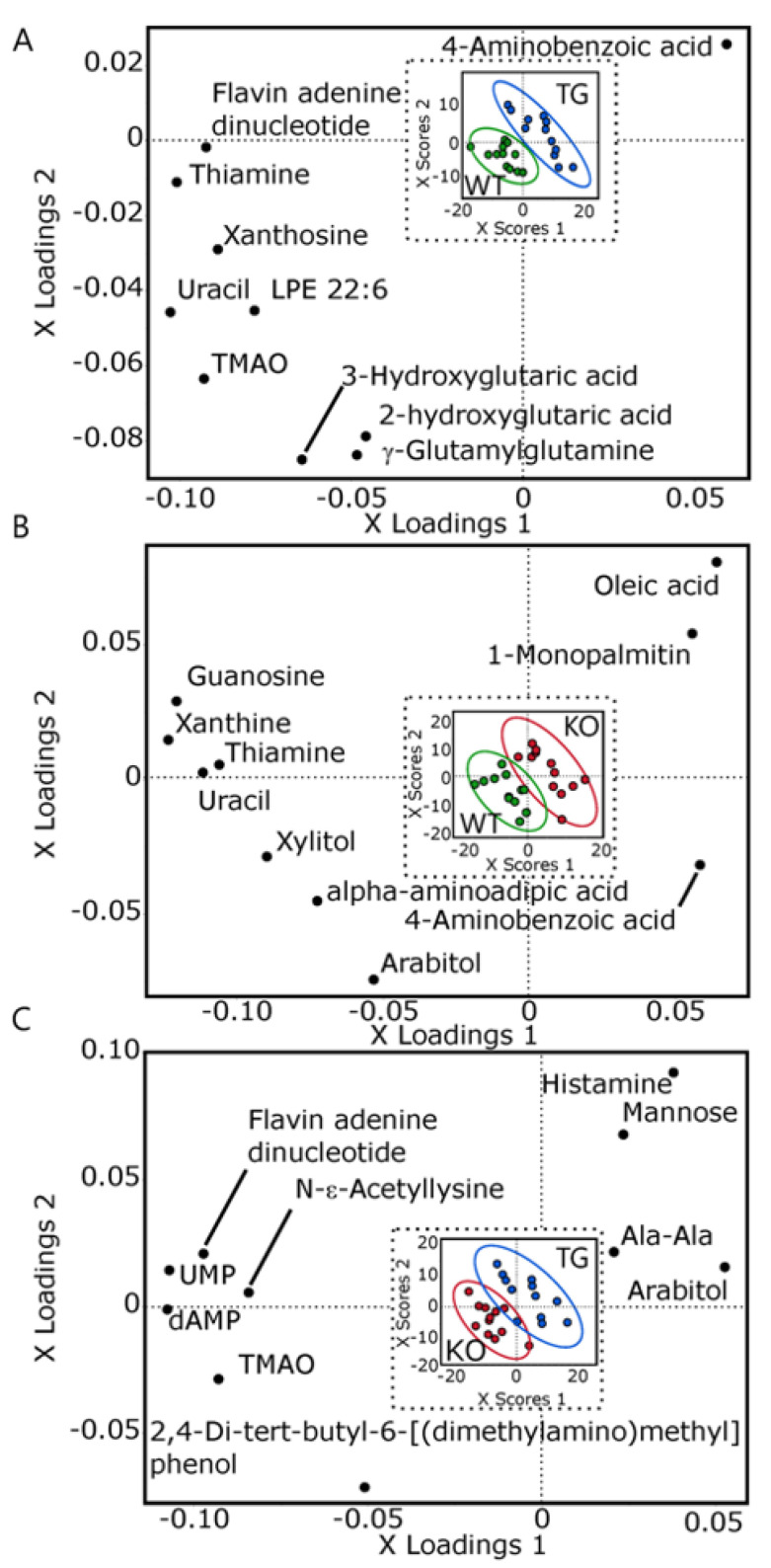
Brain metabolomic partial least square discriminant analysis of wildtype (WT), Slc19a3^−/−; SLC19A3int^ (TG) and Slc19a3^−/−^ (KO) mice (*n* = 12/grp) with equivalent plasma thiamine concentrations. Loadings plots of discriminant variables and scores plots of individual mice (insets) are shown for the following three comparisons: (**A**) WT vs. TG; 5 factors; root mean PRESS = 0.612, Q^2^ = 0.97; R^2^X = 0.551; R^2^Y = 0.998; (**B**) WT vs. KO; 5 factors; root mean PRESS = 0.746, Q^2^ = 0.867; R^2^X = 0.503; R^2^Y = 0.998; (**C**) KO vs. TG; 4 factors; root mean PRESS = 0.884, Q^2^ = 0.334; R^2^X = 0.494; R^2^Y = 0.986. Loadings plots display the metabolites with the top ten VIP scores for simplicity. Scores plot ellipses represent the 95% sample density. Green = WT; Blue = TG; Red = KO. Ala—alanine; LPE—lysophosphatyidylethanolamine; TMAO—trimethylamine oxide; UMP—uridine monophosphate; dAMP—deoxyadenosine monophosphate.

Three PLSDA analyses were then used to find specific metabolite differences between brains of the three groups of mice. Both WT vs. TG and WT vs. KO had cumulative Q^2^ > 0.85 and good separation in two-factor scores plot (Figure 3A,B). Consistent with the ChemRICH analysis, KO vs. TG only had cumulative Q^2^ of 0.33 showing incomplete separation of the two groups, with overlapping sample density in a two-factor scores plot (Figure 3C).

**Figure 4:**
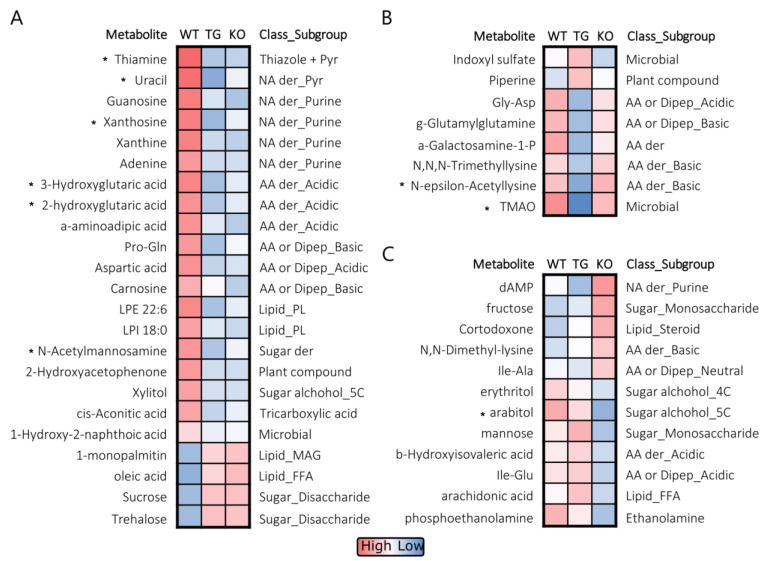
Heatmap of group mean relative intensities for the top differentiating brain metabolites among wildtype (WT), Slc19a3^−/−; SLC19A3 int^ (TG), and Slc19a3^−/−^ (KO) mice (*n* = 12/grp) with equivalent plasma thiamine concentrations. A total of 43 metabolites with partial least square discriminant analysis—random forest ensemble (PLSDA-RF) scores >1.25 in at least one of the three comparisons of WT vs. TG, WT vs. KO, and KO vs. TG are shown. Group mean relative intensities of mean centered data were used to scale heatmaps which were segregated into panels grouped by genotype comparisons: (**A**) WT most different from TG and KO; (**B**) TG most different from WT and KO, and (**C**) KO most different from WT and TG. Asterisks (*) indicates metabolites with a PLSDA-RF scores >1.25 in two of the three comparisons. Abbreviations: 4C—four carbon length; 5C—five carbon length; AA—amino acid; der—derivative; Dipep—dipeptide; FFA—free fatty acid; MAG—monoacylglyceride; NA—nucleic acid; P—phosphate; PL—phospholipid; Pyr—pyrimidine.

In general, strong discriminating variables in RF were also identified as top hits by PLSDA, with RF *p*-values < 0.05 correlated with VIP scores (WT vs. TG, R = 0.72, *p* < 0.001; WT vs. KO, R = 0.63, *p* < 0.001; KO vs. TG, R = 0.7, *p* < 0.001). For WT vs. TG and WT vs. KO comparisons, metabolites with RF *p*-values < 0.05 had VIP score > 1.25. Variable selection outputs of PLSDA and RF for each of the comparisons are shown in Supplementary Tables S4–S6. Metabolites with PLSDA-RF ensemble scores > 1.25 in any of the three comparisons are shown as heatmaps in Figure 4A–C where metabolites were grouped based on the genotype that was most different among the three groups. As expected, thiamine was reduced in both TG and KO mice (Figure 4A). Similar to the ChemRICH results, nucleic acid derivatives (such as uracil, guanosine, xanthosine) and glutarates (such as 2- and 3-hydroxyglutaric acid) were higher while disaccharide (sucrose, trehalose) were lower in WT than TG and KO. Nine metabolites had PLSDA-RF scores > 1.25 in two of the three comparisons (Supplementary Figure S5A–C). Besides the previously mentioned metabolites, N-acetylmannosamine was high in WT, trimethylamine N-oxide (TMAO), and N-ε-acetyl lysine were low in TG, while arabitol was low in the KO group. 

## 4. Discussion

Drug–nutrient interactions on thiamine status could be an important consideration when prescribing drugs that are inhibitors of thiamine transporters, such as trimethoprim and fedratinib [29,32]. While these inhibitors could reduce thiamine intestinal absorption, such problems could be mitigated by staggering drug and thiamine intake. More insidious, a drug at steady state can potentially result in sustained inhibition of thiamine distribution into peripheral tissues. These tissue-specific deficiencies could unfortunately remain obscure when RBC TDP is used as a thiamine status biomarker if RBCs and tissues are governed by different transport mechanisms. The known thiamine transporters have different tissue distributions and their roles in maintaining thiamine status are poorly defined. Understanding the contribution of THTR2 to these processes will highlight the potential impact of THTR2 inhibitors and genetic anomalies on thiamine status in a tissue-specific manner. To investigate the extraintestinal functions of THTR2, we used *Slc19a3*^−/−^ (KO) and *Slc19a3*^−/−-; *SLC19A3*int^ (TG) mouse models to probe thiamine status in various tissues. Since the TG model expresses the human THTR2 only in the intestine and maintains normal circulating thiamine, the model presents an opportunity to examine the influence of THTR2 on thiamine status in multiple organs of the mouse.

Intestinal *SLC19A3* expression rescued the low plasma thiamine levels found in KO mice under the standard thiamine diet. As previously reported [47], the KO mice did not survive on the 17 mg/kg thiamine HCl diet but were sustained by a 85 mg/kg thiamine HCl diet. Consistent with this observation, we found that intestinal THTR1 expression was unaffected by THTR2 ablation as previously reported [15]. With the insertion of intestinal *SLC19A3*, the TG mice survived on a standard thiamine diet and had normal plasma thiamine.

The THTR2 deficiency had either no discernible or mild effects on several tissues. Among the tissues measured, retroperitoneal fat had the lowest concentrations for all three thiamine vitamers, and no difference among the five experimental groups. While THTR2 mRNA is high in fat [7], the Human Protein Atlas did not detect the THTR2 protein [7], and absent or low translation may explain the lack of observed difference. In contrast, the heart had high TDP levels and TDP/thiamine ratios to support the high rates of ATP turnover in this tissue [48,49]. Despite this, the loss of THTR2 showed little impact on the heart. While no difference in cardiac vitamers was detected among the groups when compared directly, mixed models suggest that THTR2 ablation reduced heart TDP by ~8 nmol/g (27 % of WT) when plasma thiamine was accounted for. However, previous reports noted that THTR2 KOs lack obvious lesions in the heart, while showing damage to the liver and kidney [15].

In the current study, the TG mice on the 5× diet had slightly lower hepatic thiamine compared to WT but no difference between groups on the 1× diet. The role of THTR2 in maintaining hepatic thiamine status appears to be minor as compensation by other transporters largely overcame its deficit in the current mouse model. The nonspecific cation transporter OCT1 is highly expressed in the liver and may account for this protection [10]. However studies on the role of OCT1 have conflicting results with one study reporting reductions in hepatic TMP and TDP in OCT1 knockout mice [10], while another showed no impact on total hepatic thiamine vitamer levels [12]. Our results support a network of cation transporters working together to maintain thiamine vitamer status in the liver. In kidneys, the THTRs facilitate reabsorption in the renal proximal tubule while OCTs and MATES secrete thiamine into the urinary filtrate [13,50]. In mice with equivalent circulating thiamine concentrations, the three renal vitamers did not differ between the KO, TG, and WT mice, suggesting the loss of THTR2 did not directly impact kidney levels. THTR2 ablation was previously shown to upregulate renal THTR1 mRNA for compensation [15].

Among the investigated organs, the brain was the most impacted by the loss of THTR2. As brain thiamine uptake and turnover rates are similar, the organ has a low thiamine reserve in the case of inadequacy [51]. Compared to the WT on the 1× diet, the TG mice on the 1× diet achieved only 30% thiamine and 53% TMP, while the KO group had 34% thiamine and 54% TMP, indicating that THTR2 is critical for maintaining the brain thiamine pool. In the brain, both THTR2 and THTR1 are expressed on the blood–brain barrier with THTR2 located on the basement membrane and THTR1 on the luminal side of blood vessels [52]. However, unlike THTR2, THTR1 was not detected in the neurons in the cerebral cortex where the tissue was sampled [52]. Moreover, THTR1 mRNA expression was not upregulated in the brain of these mice, and the OCT1 and OCT2 double knockout had normal brain thiamine [12], suggesting that these transporters have minor roles in maintaining thiamine levels in the cerebral cortex. 

Similar to THTR2 ablation, administration of the THTR2 inhibitor trimethoprim also reduced brain thiamine. Our recent study investigating the impact of trimethoprim on thiamine disposition showed that elevations in plasma thiamine was accompanied by a decline in hepatic thiamine (*p* = 0.067) [53], likely through a combined effect on OCT1 and THTR2. Importantly, the changes in tissue thiamine despite normal circulating levels indicates that plasma and RBC thiamine status markers may not reflect tissue status when transporter function is compromised. Interestingly, despite reductions in thiamine levels due to THTR2 ablation or the administration of trimethoprim, TDP levels were not affected. Previous studies suggest that changes in plasma and brain thiamine do not result in linear changes in brain TDP [54,55] as TDP levels are regulated in response to thiamine availability and energy demand [4,56].

To probe the impact of THTR2 loss on brain metabolism, we compared metabolomic profiles from the cerebral cortex of WT, TG, and KO mice with equivalent plasma thiamine concentrations. The results showed reduced nucleic acid and amino acid derivatives, possibly due to various mechanisms including TDP-independent functions of thiamine or its derivatives, as well as other metabolic consequences resulting from alterations in endogenous THTR2 substrate concentrations. Though the metabolic functions of some thiamine derivatives, such as thiamine triphosphate (TTP) and adenosine TTP, are poorly characterized, changes in these thiamine products are unlikely to explain the observed metabolite profile changes as they are derived from TDP [57]. In addition to these derivatives, thiamine allosterically regulates malate dehydrogenase, glutamate dehydrogenase, and pyridoxal kinase, and these regulatory actions could be negatively impacted in the TG and KO brain [58]. Endogenous pyrimidines and the purines were reduced, suggesting THTR2 may also transport compounds of similar structures as thiamine, such as nucleobase derivatives. Given that TDP levels were unaltered, reductions of acidic amino acid derivatives including 2- and 3-hydroxyglutarate and aminoadipic acid are less clear but potentially linked to shifts in metabolism. Of the observed brain metabolic differences between the TG mice and the other two groups, the most notable was the substantial decrease of TMAO, a microbial metabolite linked to cardiovascular disease [59]. Further evaluations are needed to determine how the gain of intestinal THTR2 might influence trimethylamine metabolism and if this change is systemic, tissue-specific, or related to changes in the liver or gut in this unique mouse model.

Future studies should consider the benefits and drawbacks of utilizing this novel TG model. Although this model can be used to examine the impact of oral THTR2 inhibitors on intestinal thiamine absorption, it may not be optimal for evaluating the effects of these drugs on tissue status beyond the intestines. Additionally, the ramifications of the low brain thiamine levels should be investigated in the TG mouse subjected to physical, psychological, and/or hypermetabolic states. Since higher metabolic states increase thiamine demand, these animals may be less resilient during metabolic stress. Notably, clinical THTR2 dysfunction from various mutations in *SLC19A3* results in the rare biotin–thiamine-responsive basal ganglia disease. This disorder is distinguished by stress-triggered episodic encephalopathy, dystonia, ophthalmoplegia, and seizures, which can prove fatal without improvement [17]. While three subtypes of this disease are recognized, lesions of the basal ganglia, thalamus, cortex, and brainstem are commonly seen. Moreover, response to the high thiamine supplement treatment depends on the specific gene mutation. As these patients have reduced or absent THTR2 activity and may not be able to upregulate THTR2, their stress response is compromised [60]. Further research should address the impacts of THTR2 inhibitors on reduced brain thiamine levels, metabolic response to stress, and their potential to exacerbate thiamine deficiency symptoms. 

Our study has a few limitations. The brains of the TG mice were profiled at ~21 weeks and the mice themselves were kept no longer than 8 months so developmental and long-term effects of reduced brain thiamine in the TG model are unknown and may include compensatory mechanisms or a health deterioration over time. While previous investigation on the KO model showed premature death at one year [15], it is unclear if the changes in brain thiamine would result in a similar fate in the TG mice. Secondly, translatability to humans is not clear due to species differences. Although the ratio of plasma thiamine (nM) to brain concentration (nmol/g tissue) in WT mice were similar to those reported in humans [49], rodents have greater metabolism, with ~10x more thiamine in plasma and ~23x more thiamine in brain than humans. Based on human’s lower plasma thiamine concentration (~10 nM), OCT1 lower affinity for thiamine [10,12,61,62], and lower expression in certain tissues compared to mouse [63], OCT1 may overemphasize OCT1 thiamine transport in humans [61]. Thus, it is possible that other thiamine transporters including THTR2 may be underestimated in mice when translating to humans.

## 5. Conclusions

In conclusion, our findings suggest that THTR2 is irreplaceable for maintaining brain thiamine levels. As a significant transporter for thiamine distribution, THTR2 should be taken into consideration for drug–nutrient interactions, not only for gut absorption and renal elimination, but also uptake into tissues, especially the brain where inhibition can lead to localized deficiency and altered metabolism.

## Figures and Tables

**Table 1:** Impact of genotype, plasma thiamine, and sex on tissue vitamer levels revealed by mixed effect modeling using WT (*n* = 12) and TG (*n* = 12) mice from both diets.

Tissue	Vitamer	β Coefficient ^a^		
		Genotype [TG]	Plasma Thiamine	Sex [F]
RBC	TDP		0.007 **	
Duodenum	Thiamine	0.488 ***	0.009 ****	
	TMP	0.702 **	0.012 ***	
	TDP			
Brain	Thiamine	−0.55 ****		−0.109 *
	TMP	−1.04 ***		
	TDP		0.006 *	
Kidney	Thiamine		0.002 ****	
	TMP		0.001 **	
	TDP		0.059 ****	1.883 *
Liver	Thiamine	−0.108 **		
	TMP			
	TDP			
Heart	Thiamine			
	TMP			
	TDP	−4.05 *	−0.039 *	
Fat	Thiamine		0.002 **	
	TMP			
	TDP			

a—For categorical variables, positive β coefficients represent higher levels in the TG group or in females, while negative coefficients represent the inverse. * *p* ≤ 0.05, ** *p* ≤ 0.01, *** *p* ≤ 0.001, **** *p* ≤ 0.0001. No interactions terms were significant.

## Data Availability

The metabolomic dataset will be deposited in the Metabolomic Workbench. The data presented in this study are available in the Supplementary Material.

## Supplementary Materials

The following supporting information can be downloaded at: https://www.mdpi.com/article/10.3390/metabo13080885/s1, Supplementary Information: Thiamine vitamer assays performance; Figure S1: Expression of hTHTR2, mTHTR2, and mTHTR1 in wildtype (WT), Slc19a3^−/−;intestinal SLC19A3^ (TG), and Slc19a3^−/−^ (KO) mice; Figure S2: Characterization of the five groups of mice; Figure S3: Brain thiamine and TDP measured from mice given 2 mg/kg thiamine (*n* = 6) or combination 2 mg/kg thiamine and 61.5 mg/kg trimethoprim (*n* = 4) via oral gavage; Figure S4: Impact of standard or 5× standard dietary thiamine on thiamine vitamers in tissue and blood compartments of wildtype (WT), Slc19a3^−/−;intestinal SLC19A3^ (TG) Slc19a3^−/−^ (KO) mice (*n* = 6/grp); Figure S5: Nine top-hit brain metabolites that were different in wildtype (WT), Slc19a3^−/−;intestinal SLC19A3^ (TG) and Slc19a3^−/−^ (KO) mice with equivalent plasma thiamine concentrations; Table S1: Precision of sample replicates; Table S3: Linear regression modeling vitamers as a function of genotype, diet, and sex; Table S4: PLSDA and RF outputs of brain metabolites for WT and TG comparison; Table S5: PLSDA and RF outputs of brain metabolites for WT and KO comparison; Table S6: PLSDA and RF outputs of brain metabolites for KO and TG comparison.

## Appendix A

### Thiamine Vitamer Assays Performance

In general, background levels in method blanks were low. For the plasma thiamine assay, blanks values were <30% of the lowest thiamine concentration and pooled mouse plasma replicates had a coefficient of variation (CVs) of <10% with 268 ± 14 nM (mean ± SD). For the RBCs analysis, blanks were <10% of the lowest TDP concentration, and RBC batch triplicates had TDP CVs of 7.5%. Brain, liver, heart, and kidney analyses were performed in two batches, with blank values for thiamine, TMP and TDP being 14.1–35.2%, 1.0–1.7%, and <1% of the lowest reported samples, respectively. For the adipose tissue batch, blank values were 69.7% for thiamine, 19.4% for TMP and 18.3% for TDP of the lowest sample. For the duodenum tissue batch, blanks values were 11.4% for thiamine, 6.9% for TMP and 8.8% for TDP of the lowest sample. Replicate data were variable (Supplementary Table S1) and may be due to heterogeneity in tissues as the tissues were not homogenized prior to sample weighing. Prior attempts to homogenize and pool tissues tended to increase thiamine and TMP levels and decrease TDP levels.
