# Supplementary material for: The Impacts of Slc19a3 Deletion and Intestinal SLC19A3 Insertion on Thiamine Distribution and Brain Metabolism in the Mouse
Source: Metabolites. 2023 Jul 26;13(8):885. doi: 10.3390/metabo13080885 (PMC10456376; doi:10.3390/metabo13080885)
Supplement: Supplementary file 1 [file metabolites-13-00885-s001.zip › 2022 ThiamineDistrib_SuppFig V1.pdf]

Supplemental Figures

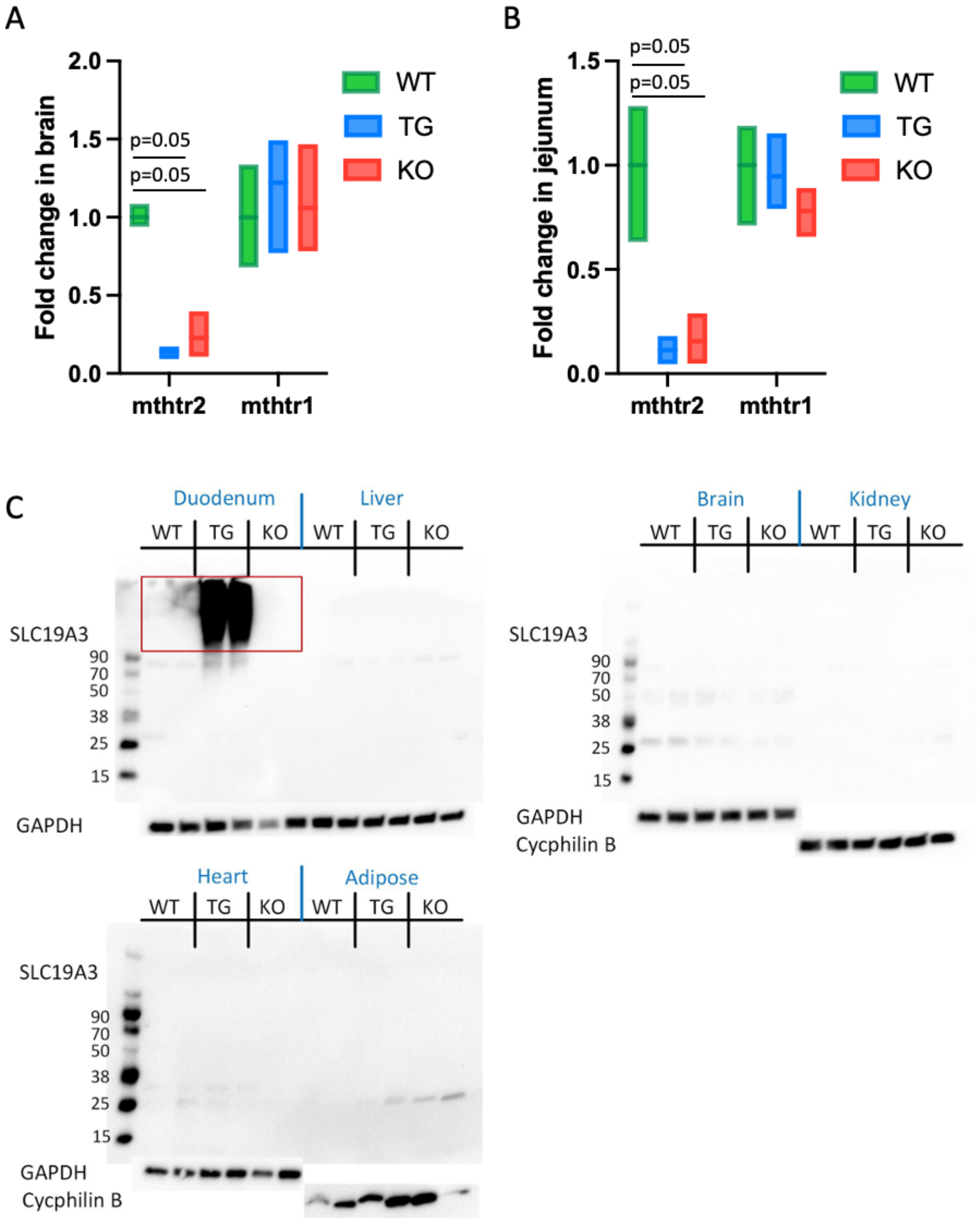

Supplementary Figure S1. Expression of hTHTR2, mTHTR2, and mTHTR1 in wildtype (WT), $Slc19a3^{-/-;intestinal\ SLC19A3}$ (TG) and $Slc19a3^{-/-}$ (KO) mice. Fold change in RNA expression of mTHTR2 and mTHTR1 with nonparametric Mann-Whitney U tests for **A**) brain and **B**) jejunum tissues. **C**) Western blots for hTHTR2 in duodenum, liver, brain, kidney, heart, and adipose tissue.

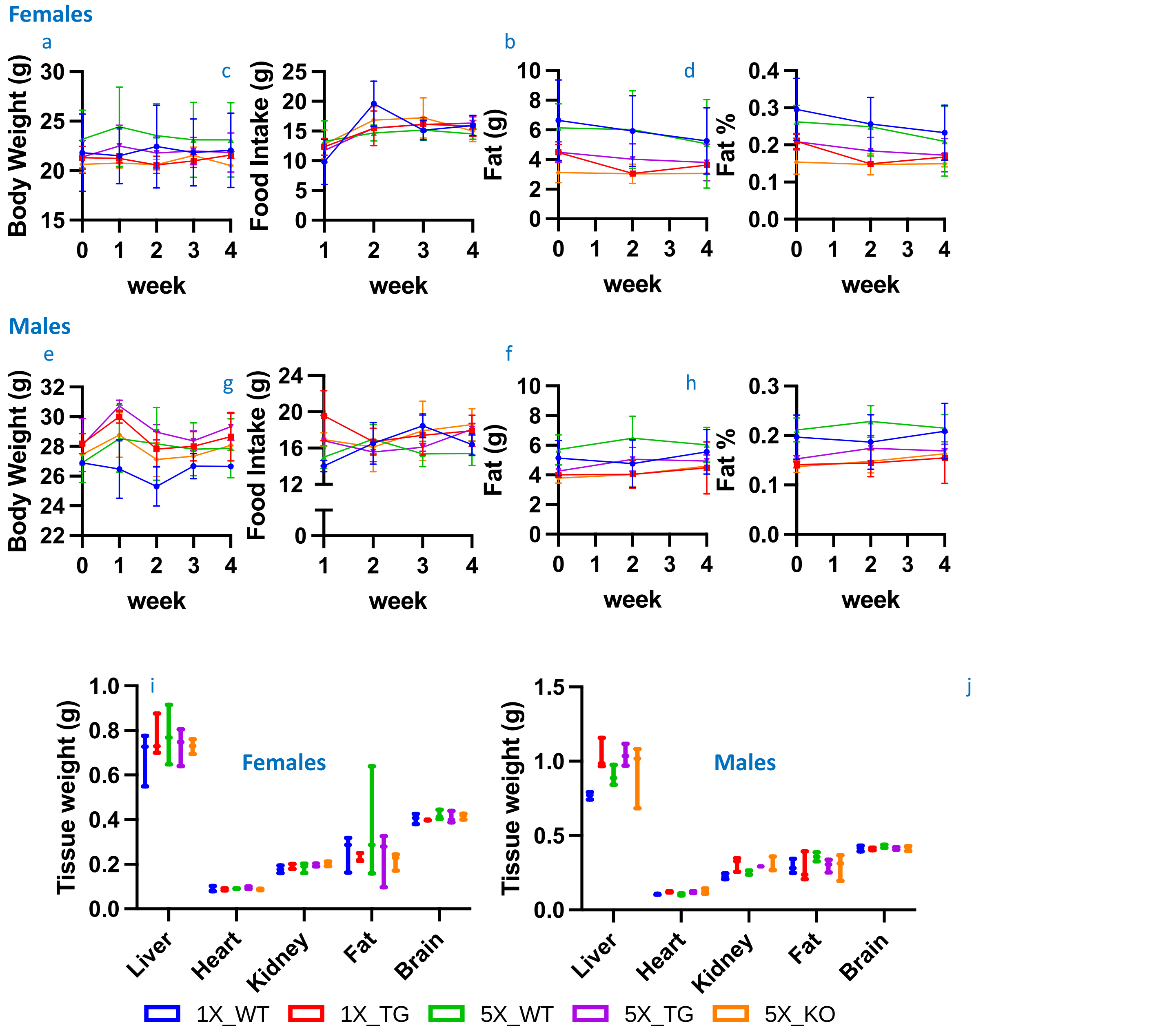

Supplementary Figure S2. Characterization of the five groups of mice. Body weight, food intake, retroperitoneal fat mass, and fat percent composition were monitored, and various tissue weights were recorded for females (a-d) and males (e-h). Various tissue weights at the end of the study were recorded for females (i) and males (j).

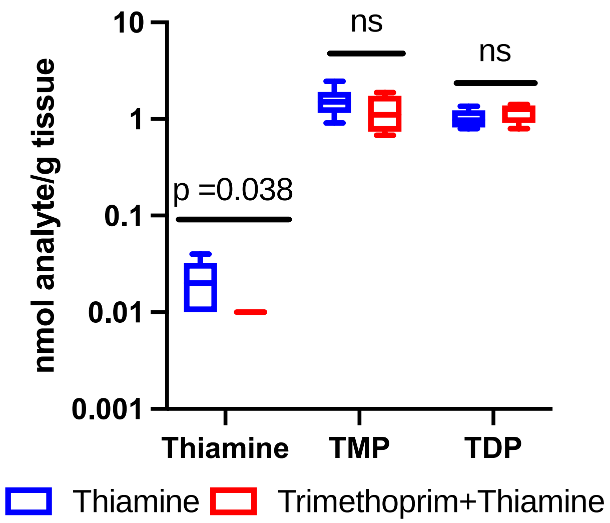

Supplementary Figure S3. Brain thiamine and TDP measured from mice given 2mg/kg thiamine (n=6) or combination 2mg/kg thiamine and 61.5mg/kg trimethoprim (n=4) via oral gavage. Brain tissue was collected 3h post treatment. Wilcoxon rank sum exact test was performed to test difference between groups.

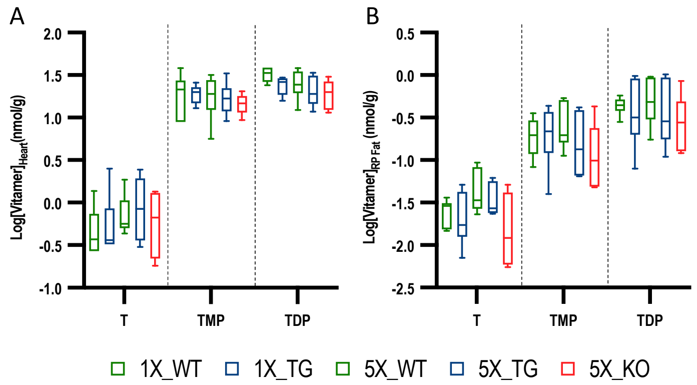

Supplementary Figure S4. Impact of standard or 5x standard dietary thiamine on thiamine vitamers in tissue and blood compartment of wildtype (WT), Slc19a3$^{-/-;\text{intestinal SLC19A3}}$ (TG) Slc19a3$^{-/-}$ (KO) mice (n =6/grp). A) heart levels and **B**) retroperitoneal fat levels. To compare the five groups, ANOVA with post hoc Tukey tests were performed for each vitamer. Means not sharing letter annotations differ between groups. One plasma sample in the 5X_WT group was excluded from plots. T= Thiamine; TMP = thiamine monophosphate; and TDP = thiamine diphosphate.

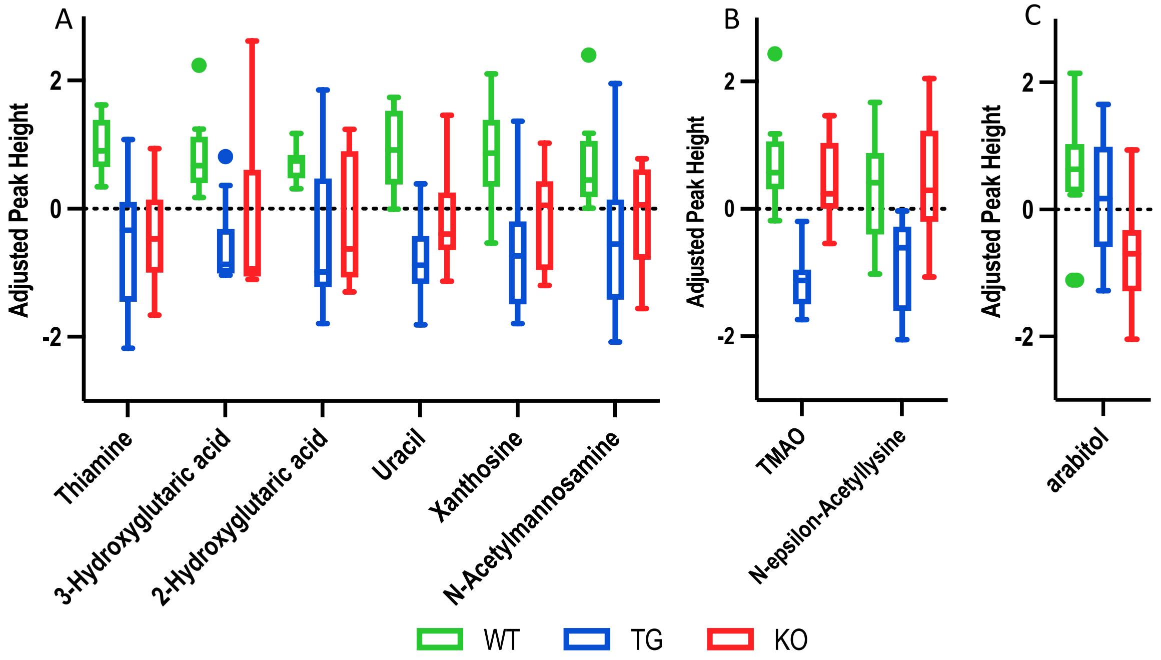

Supplementary Figure S5. Nine top-hit brain metabolites that were different in wildtype (WT), Slc19a3$^{-/-;intestinal\ SLC19A3}$ (TG) and Slc19a3$^{-/-}$ (KO) mice with equivalent plasma thiamine concentrations. Metabolites with partial least square discriminant analysis – random forest ensemble score (PLSDA-RF score >1.25) in two of the three comparisons (WT vs TG, WT vs KO, and KO vs TG) are plotted in which **A)** WT, **B)** TG, and **C)** KO groups differ from the other two groups.
